# ECM Composition Differentially Regulates Intracellular and Extracellular pH in Normal and Cancer Pancreatic Duct Epithelial Cells

**DOI:** 10.3390/ijms241310632

**Published:** 2023-06-25

**Authors:** Daria Di Molfetta, Stefania Cannone, Maria Raffaella Greco, Rosa Caroppo, Francesca Piccapane, Tiago Miguel Amaral Carvalho, Concetta Altamura, Ilaria Saltarella, Diana Tavares Valente, Jean Francois Desaphy, Stephan J. Reshkin, Rosa Angela Cardone

**Affiliations:** 1 Department of Biosciences, Biotechnology and Environment, University of Bari Aldo Moro, 70126 Bari, Italy; daria.dimolfetta@uniba.it (D.D.M.); stephanjoel.reshkin@uniba.it (S.J.R.); 2 Department of Biomedical Sciences and Human Oncology, School of Medicine, University of Bari Aldo Moro, 70124 Bari, Italyjeanfrancois.desaphy@uniba.it (J.F.D.); 3 Life and Health Sciences Research Institute (ICVS), School of Medicine, University of Minho, Campus de Gualtar, 4710-057 Braga, Portugal

**Keywords:** PDAC, NHE1, bicarbonate transport

## Abstract

Intracellular pH (pHi) regulation is a challenge for the exocrine pancreas, where the luminal secretion of bicarbonate-rich fluid is accompanied by interstitial flows of acid. This acid–base transport requires a plethora of ion transporters, including bicarbonate transporters and the Na^+^/H^+^ exchanger isoform 1 (NHE1), which are dysregulated in Pancreatic Ductal Adenocarcinoma (PDAC). PDAC progression is favored by a Collagen-I rich extracellular matrix (ECM) which exacerbates the physiological interstitial acidosis. In organotypic cultures of normal human pancreatic cells (HPDE), parenchymal cancer cells (CPCs) and cancer stem cells (CSCs) growing on matrices reproducing ECM changes during progression, we studied resting pHi, the pHi response to fluxes of NaHCO_3_ and acidosis and the role of NHE1 in pHi regulation. Our findings show that: (i) on the physiological ECM, HPDE cells have the most alkaline pHi, followed by CSCs and CPCs, while a Collagen I-rich ECM reverses the acid–base balance in cancer cells compared to normal cells; (ii) both resting pHi and pHi recovery from an acid load are reduced by extracellular NaHCO_3_, especially in HPDE cells on a normal ECM; (iii) cancer cell NHE1 activity is less affected by NaHCO_3_. We conclude that ECM composition and the fluctuations of pHe cooperate to predispose pHi homeostasis towards the presence of NaHCO_3_ gradients similar to that expected in the tumor.

## 1. Introduction

The most common pancreatic cancer is Pancreatic Ductal Adenocarcinoma (PDAC), which is also one of the deadliest of human cancers. PDAC arises in the smaller-caliber ducts in the exocrine compartment of the pancreas [1,2]. During digestion, normal ductal pancreatic cells secrete an alkaline (pH ≈ 8–8.5), NaHCO_3_-rich fluid [3,4] to neutralize the acidic chyme arriving from the stomach [5]. Alkaline secretion leads to parallel acidification of the pancreatic interstitium [6]. Therefore, due to the alternation between of the digestive and the resting phases, the ductal cells are exposed to variable gradients of extracellular pH (pHe) [7]: alkaline on the apical side and acidic on the basolateral side, which rapidly return to resting state [8]. These pHe gradients translate into specular variations of intracellular pH (pHi), whose homeostasis is ensured by a complex plethora of ion transporters/channels such as: Cl^−^/HCO_3_^−^ (SLC26), Na^+^/H^+^ (NHE1), Na^+^/HCO_3_^−^ (NBCs), the gastric and non-gastric H^+^/K^+^-ATPases [9], and CFTR [10,11].

It has been reported that the onset of PDAC changes the expression or the activity of pH-regulatory transporters [6], favoring the reversed acid–base balance typical of cancer cells [12,13,14,15,16]. Indeed, in a data mining study of gene expression profiles of three databases comparing normal tissues with PDAC tissues, there was consistent up-regulation of CAIX and of the basolateral acid extruders MCT4 and the NHEs, especially NHE1 [17,18]. This altered expression could be involved in the combined alkalinization of pHi and the acidification of the tumor microenvironment (TME) [19], which in many cancers, including PDAC, reaches pHe values ranging from 6.4 to 7.0 [20,21]. While this acidic pHe induces apoptosis in normal cells, it activates survival and growth in some premalignant cells, which have greater capacity to adapt to the acidic microenvironment [22]. As PDAC cells arise in an organ already exposed to highly dynamic and cyclically variable pHe landscapes [23] they have an intrinsic ability to survive and take advantage of such an acidic microenvironment, which fosters the aggressive features of PDAC, such as early metastasis, immunosuppression and resistance to immune and chemotherapy [15,24,25,26]. Indeed, acidic pHe promotes the emergence of very aggressive cancer cells with stem-like properties [27,28,29], the cancer stem cells (CSCs) [29,30], with abilities of self-renewal, metastasis and chemoresistance [31,32,33,34]. Moreover, due to the acid adaptation, tumor cells secrete collagen-producing enzymes to remodel their surrounding extracellular matrix (ECM) and construct niches in which they can survive and evolve towards malignancy [22]. Indeed, a typical feature of the PDAC TME is its prominent desmoplasia consisting in an increasingly hyperplastic connective tissue, mostly composed of stromal cells and ECM components, in particular collagen I, which is enriched in the tumor stroma as the tumor progresses [27,35,36,37]. This abundant stroma blocks the formation of a vascular meshwork [38] leading to the generation of dynamic areas of hypoxia and low nutrients [39], which boost PDAC pathophysiology. However, how ECM composition interacts with extracellular changes of NaHCO_3_ and fluxes of acidosis to regulate pHi in normal and tumor cells, including the CSCs, is still unknown. Here, we used a 3D organotypic culture model mimicking the changing ECM composition from the pre-malignant pancreas to an early/invasive tumor (both Matrigel rich) to the more developed PDAC (collagen-I rich) stroma [37,40,41,42,43,44,45] to evaluate the effect of the different ECMs on the ability of normal Human Pancreatic Duct Cells (HPDE), Panc-1 Cancer Parenchymal Cells (CPCs), and their derived Cancer Stem Cells (CSCs) to regulate pHi, when both were exposed to various pHes and NaHCO_3_ concentrations, which reproduce the oscillating extracellular conditions cyclically occurring in the pancreas. We also measured the physio-pathological role of one of the principal pHi regulators, NHE1, in the maintenance of pHi homeostasis when the above cell types were exposed to these transient pHe/ NaHCO_3_ changes on the different normal/tumor ECMs. Our data indicate that the ECM composition actively drives (i) the resting pHi in normal cells, the Panc1 tumor cells and their derived CSCs, (ii) the pHi response of these cells to the presence of NaHCO_3_ in both neutral and acidic pHe, (iii) their pHi recovery from an intracellular acid load, and (iv) their NHE1 activity. Altogether, these findings suggest a scenario in which the ECM composition cooperates with the fluctuations of pHe to regulate both pHi homeostasis and the activity of NHE1, which is a known promoter of both tumor initiation and malignant progression in several tumor types, including PDAC.

## 2. Results

The experiments were performed using a 3D culture system that models the in vivo changes in ECM composition occurring during PDAC progression [40,41,44,46]. For this, we used three different stepwise mixes of Matrigel (M) and Collagen I (C)-based matrices (90M-10C; 70M-30C; 10M-90C), mimicking the spatio-temporal progression from the normal basement membrane (90M) to the increasing stromal reaction (70M and 10M) associated with PDAC development [21,47,48,49,50]. As stromal Collagen I increases during progression from a normal pancreas to PDAC, we grew the normal HPDE cells exclusively on ECMs modeling the transition from a normal ECM to an early-stage disease ECM (90M-10C and 70M-30C, respectively) while we also cultured the tumor CPCs and CSCs on a third, more fibrotic ECM (10M-90C), representative of the most advanced PDAC. Furthermore, to mimic the cycle of digestive hormone-stimulated secretion of NaHCO_3_ and the subsequent proton release, followed by the resetting of basal conditions, we perfused the cells with Ringers, half of which contained NaHCO_3_. Perfusing the cells with NaHCO_3_ recreates the extracellular environment presented during the secretion of the alkaline fluid in the pancreatic duct, which provides the substrate necessary for the activation of Na/HCO_3_ transporters, which are highly expressed in normal pancreatic duct cells [32].

### 2.1. ECM Composition, Extracellular NaHCO_3_ and Acidic pHe Affect Resting pHi in Normal and Tumor Cells

We analyzed the effects of the ECM composition on resting pHi in either the presence or the absence of extracellular NaHCO_3_ in the normal pancreatic ductal cell line, the HPDE cells, and in the tumor cell CPCs and CSCs. The normal HPDE cells were grown on 90M-10C and 70M-30C, while the tumor CPCs and CSCs were cultured on a third, more fibrotic ECM (10M-90C), representative of the most advanced PDAC. Moreover, in each of these growth conditions, resting pHi was measured at both pHe 7.4 and when cells were exposed to transient fluxes of interstitial acidosis (pHe 6.7), biomimetic of both the pancreatic, periodic pHe oscillations occurring during digestion and the transient intratumoral acidic areas. For this, a spectrofluorometer equipped with a gravity flow-controlled perfusion system permitting an alternating cell perfusion with Ringer with and without NaHCO_3_, buffered at both pHe 7.4 and pHe 6.7, was used. The cells, grown on the different ECMs, were loaded with the pH-sensitive probe BCECF-AM, inserted in the cuvette and alternatively perfused with the Ringers at both pHes in order to obtain a dynamic measurement of their pHi in response to the changing pHes and the presence of NaHCO_3_.

**Figure 1:**
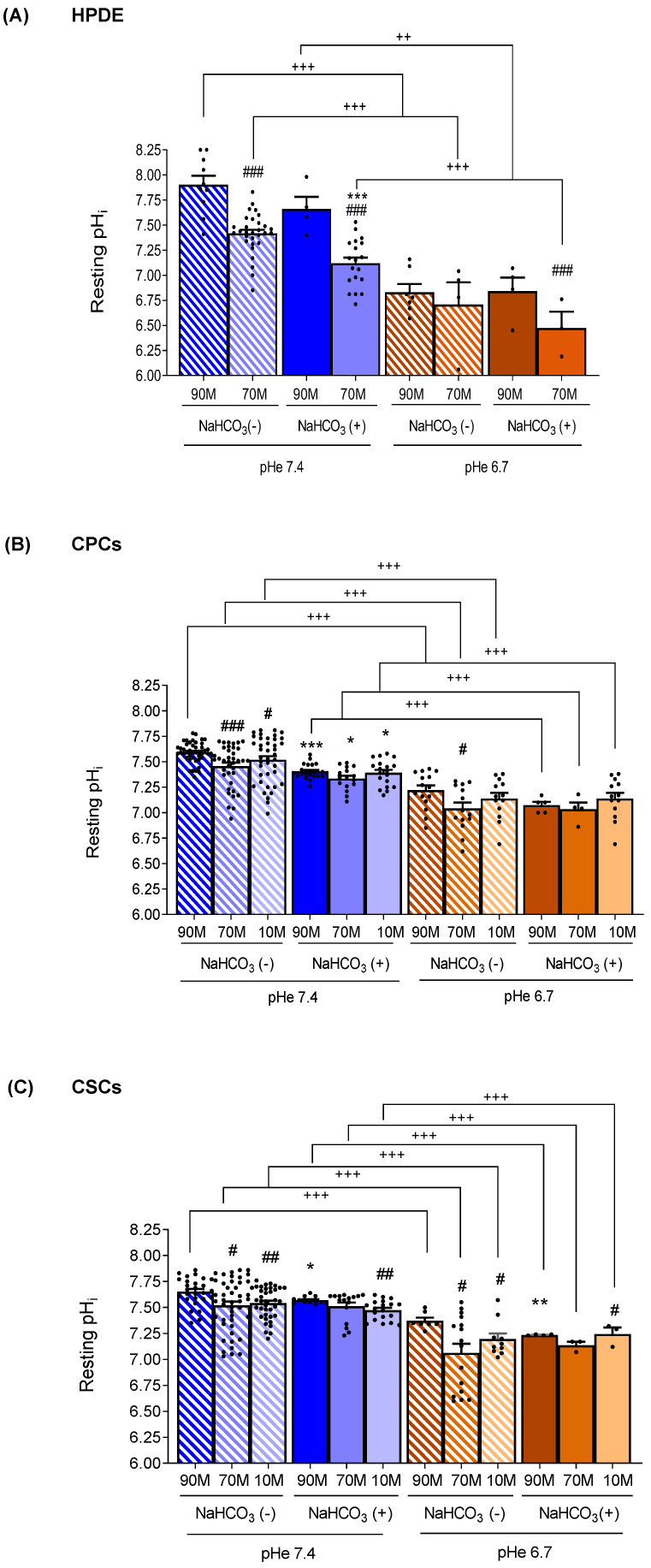
Effect of the ECM composition (from a less fibrotic to a more fibrotic ECM) and different pHe (pHe 7.4 and pHe 6.7) in the presence (NaHCO_3_ (+)) or absence of NaHCO_3_ (NaHCO_3_ (−)) on resting pHi. The cells were alternatively perfused with Ringer with and without NaHCO_3_ (see Supplementary Table S1) at both pHes to determine the effect of the changing pHe and the presence or absence of NaHCO_3_ on resting pHi. (a) The normal HPDE cells (**A**) were grown on a matrix resembling the normal pancreatic ECM (90M-10C) and a low fibrotic ECM (70M-30C), while the tumor cells (**B**) CPCs and (**C**) CSCs, concordant with the increasing level of desmoplasia in fully established PDAC, were also grown on a highly fibrotic ECM (10M-90C). Significance between groups: # *p* < 0.05; ## *p* < 0.01; ### *p* < 0.001 compared to 90M in the same NaHCO_3_/pHe conditions; * *p* < 0.05; ** *p* < 0.01; *** *p* < 0.001 comparing NaHCO_3_ (+) versus NaHCO_3_ (−) on the same ECM/pHe; ++ *p* < 0.01; +++ *p* < 0.001.

We started by comparing the resting pHi values in the HPDE, CPCs and CSCs growing on the different ECMs, first when exposed at pHe 7.4 and then at pHe 6.7. We found that in a physiological ECM composition (90M-10C) and at pHe 7.4, the normal HPDE cells (Figure 1A) were more alkaline than either the CPCs or CSCs (Figure 1B,C), independently of the presence of extracellular NaHCO_3_. Importantly for the tumor cells growing on 90M-10C, the CSCs were more alkaline than the CPCs. This is in accordance with both their higher aggressiveness and trans-differentiation ability towards a vascular-like phenotype and with their chemoresistant characteristics that drive tumor progression [21,32] However, in line with the well-known reversed acid–base balance of cancer cells [13,51,52], in tumor-like ECMs (70M-30C and 10M-90C) and especially at the tumor-like acidic pHe (pHe 6.7), the cancer cells were much more alkaline than the normal cells. Indeed, of the three cell lines, cell perfusion with Ringers buffered at pHe 6.7 produced the strongest cytosolic acidification in the HPDE cells (Figure 1A), with an acidification of approximately 0.9 pH units in both the absence and presence of NaHCO_3_. This intracellular acidification was especially higher when HPDE cells grew in the ECMs resembling the normal pancreatic ECM (90M-10C) compared to their growth on a more fibrotic ECM (70M-30C). Unlike HPDE cells, both the CPCs and CSCs (Figure 1B,C), when perfused at pHe 6.7, were acidified by approximately 0.3 and 0.25 pH units, respectively. This confirms that, in conditions that mimic the TME (i.e., Collagen I-rich ECMs and pHe 6.7), pHi regulation mirrors the typical pHi/pHe gradient inversion of the tumor cells compared to normal cells such that CPCs and CSCs have a more alkaline pHi than the HPDE cells.

We then analyzed the effect of NaHCO_3_ on the cells’ ability to regulate their pHi at both pHe 7.4 and acidic pHe. Interestingly, at pHe 7.4, the HPDE cells reduced their pHi more greatly in the presence of NaHCO_3_ than the cancer cells, whatever the ECM composition (Figure 1A). Moreover, the CPCs (Figure 1B) were significantly more sensitive to the presence of NaHCO_3_ than the CSCs (Figure 1C) at pHe 7.4, as the CPCs had a reduction of approximately 0.19 pH units vs. 0.08 pH units in the CSCs. Therefore, pHi regulation was especially dependent on the presence of external NaHCO_3_ in the normal HPDE cells while this NaHCO_3_ dependence became ever less pronounced with the increasing cellular aggressiveness (from CPCs to CSCs). Indeed, at pHe 7.4, the CSCs maintained a very alkaline pHi both in the presence and in the absence of NaHCO_3_ (Figure 1C). This is in line with the data showing that in pancreatic cancer cells, the NaHCO_3_ –dependent transporters are downregulated and NHE1 is overexpressed compared to normal cells [17,53] delegating pHi regulation to NaHCO_3_-independent mechanisms. Interestingly, at pHe 6.7, both HPDE cells and CPCs regulated their pHi independently of the presence of extracellular NaHCO_3_, while CSCs were sensitive to NaHCO_3_ only in the 90M-10C ECM. Lastly, we analyzed how changes in the ECM composition affected resting pHi in each cell line exposed to the different experimental conditions. We found that the ECM composition was capable of changing pHi especially in the HPDE cells at pHe 7.4 both in the presence and in the absence of NaHCO_3_ (Figure 1A). Indeed, as Collagen I was increased from 90M-10C to 70M-30C, HPDE pHi decreased by approximately 0.5 pH units independently of external NaHCO_3_. Collagen I enrichment in the ECM also reduced pHi in cancer cells, but to a much lower extent compared to HPDE cells. Furthermore, while in CPCs the pHi reduction was only detected at pHe 7.4 and in the absence of NaHCO_3_, in CSCs it occurred at both pHes regardless of the presence of NaHCO_3_.

**Figure 2:**
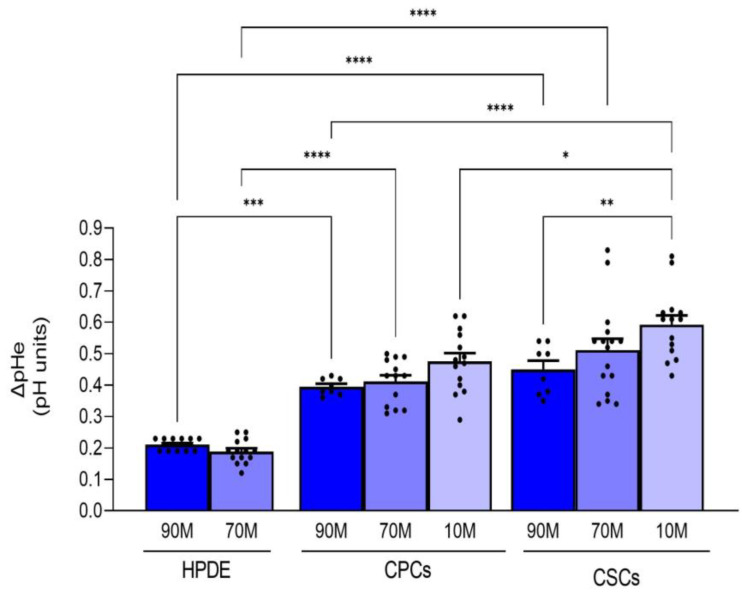
Effect of the ECM composition (from a less fibrotic to a more fibrotic ECM) on the ability of the three cell lines to acidify their extracellular NaHCO_3_-containing medium measured with single-barreled H^+^-sensitive microelectrodes. Acidification is expressed as ∆pH in the medium of confluent cells over 24 h. The acidification rate of the HPDE cells was significantly less than either the CPCs or the CSCs on all ECMs. Significance between groups: * *p* < 0.05; ** *p* < 0.01; *** *p* < 0.001 and **** *p* < 0.0001.

The effects of ECM composition on pHi dynamics in the normal cells (HPDE) and the two cancer cell types are reflected on their ability to acidify the extracellular medium (Figure 2). Indeed, the normal HPDE cells had a very low acidification capacity (∆pHe: 0.212 ± 0.005 vs. 0.181 ± 0.01 for 90M-10C vs. 70M-30C, respectively) in line with all non-cancerous cells. This already low acidification ability tended to be further decreased in the presence of higher Collagen I in the ECM, as might be expected since normal pancreatic ductal cells usually do not experience ECM with high levels of Collagen I. Importantly, the CSCs had a greater ability to acidify the extracellular medium and this was increased with the enrichment of Collagen I in the ECM, which reproduces the Collagen I-rich niche in which the CSCs reside in vivo (∆pHe: 0.446 ± 0.03 vs. 0.414 ± 0.02 on 90M-10C; 0.512 ± 0.04 vs. 0.423 ± 0.02 on 70M-30C and 0.602 ± 0.03 vs. 0.466 ± 0.03 on 10M-90C, for CSCs vs. CPCs, respectively).

### 2.2. Collagen I Enrichment in the ECM Decreases pHi Recovery Ability Mediated by NHE1 in Cancer Cells and NaHCO_3_ Transport in Normal Cells

**Figure 3:**
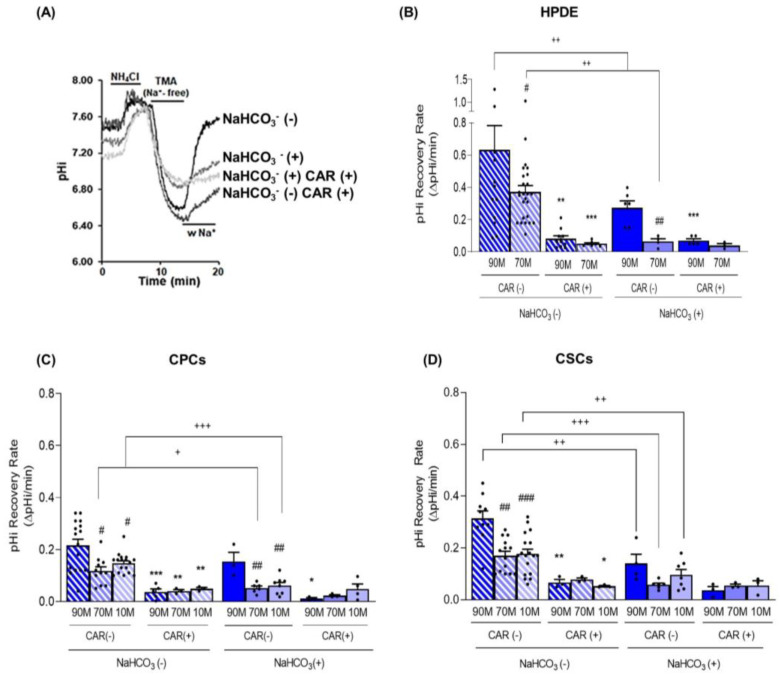
(**A**) Representative traces of the pHi recovery rate from an NH_4_Cl prepulse-induced acidification in HPDE 3D organotypic cultures labelled with the pH-sensitive probe BCECF. The experiment was performed according to the Boron protocol (please see Materials and Methods) with or without NaHCO_3_ ((NaHCO_3_ (+) or NaHCO_3_ (−), respectively), and in the absence (CAR (−)) and presence (CAR (+)) of Cariporide (5 µM) during the pH recovery to evaluate the contribution of NHE1. In both the normal HPDE cells (**B**) and in the cancer cells, CPCs (**C**) and CSCs (**D**), the pHi recovery is always dependent on NHE1 activity in the absence of NaHCO_3_. Results are presented as mean ± SEM. Significance between groups: * *p* < 0.05; ** *p* < 0.01; *** *p* < 0.001 compared to their own pHi measured without Cariporide on the same ECM; # *p* < 0.05; ## *p* < 0.01; ### *p* < 0.001 compared to 90M-10C for each cell line; + *p* < 0.05; ++ *p* < 0.01; +++ *p* < 0.001.

**Figure 4:**
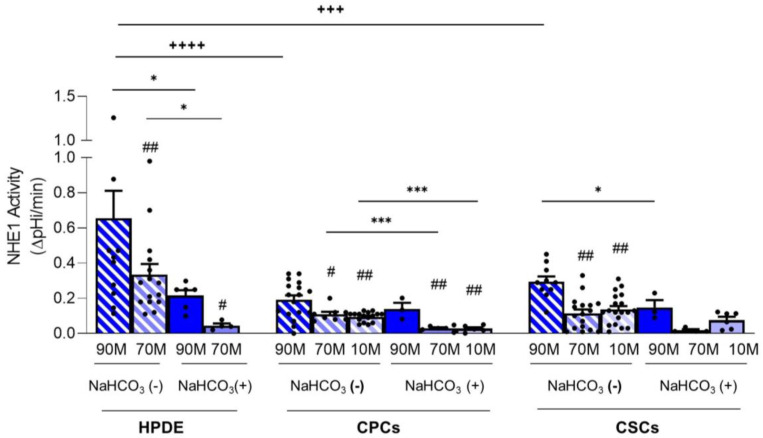
NHE1 activity calculated as the pHi recovery in control condition subtracted from pHi recovery in the presence of Cariporide for HPDE, CPC and CSC cell lines. Significance between groups: # *p* < 0.05; ## *p* < 0.01 compared to 90M-10C for each cell line; * *p* < 0.05, *** *p* < 0.001; +++ *p* < 0.001 and ++++ *p* < 0.0001.

To characterize the transport mechanisms that regulate pHi at pHe 7.4 in the three cell lines growing on the different ECMs and in the presence or absence of extracellular NaHCO_3_, we perfused cell monolayers with Ringers (pHe 7.4), half containing NaHCO_3_ and we evaluated the pHi recovery rate after an acute acid load with 40 mM NH_4_Cl, as previously described [54]. Since in most cell types, the NHE1 represents the principal pHi recovery system, NHE1 expression increases in PDAC [55] and it is involved in PDAC progression [56], we explored its contribution to pHi recovery by performing the experiments in both control conditions and in the presence of its specific inhibitor Cariporide (5 µM). Representative examples of the changes in pHi in response to an acid load followed by pHi recovery of cells perfused with either NaHCO_3_-free or NaHCO_3_-containing Ringers in the absence or presence of Cariporide are shown in Figure 3A. As reported in Figure 3B, both in the presence and in the absence of NaHCO_3_, the HPDE cells showed a much higher pHi recovery ability compared to CPCs and CSCs grown in the same experimental setup (Figure 3C,D) and this cytoplasmic re-alkalinization was largely sustained by the activity of NHE1 (see the effect of Cariporide in Figure 4). When HPDE cells growing on 90M-10C and 70M-30C were perfused with Ringer containing NaHCO_3_, their ability to recover pHi dropped by almost 83% ± 1.7 and 84.98% ± 3.1, respectively, but was still dependent on NHE1 activity only on 90M-10C (Figure 3B). Similarly, we found that both CPCs and CSCs recovered their pHi faster when perfused with NaHCO_3_-free Ringer than the in presence of NaHCO_3_ (Figure 3C,D). Moreover, in the absence of NaHCO_3_, their pHi recovery was mainly operated by NHE1 in all the ECMs, as its specific inhibition reduced the ability to recover pHi by about 80% (Figure 3C,D). On the contrary, in the presence of NaHCO_3_, pHi recovery required NHE1 activity only for the CPCs growing on 90M-10C, while in CSCs it was totally independent of NHE1 (Figure 3C,D).

To more clearly show the contribution of NHE1 to pHi recovery in the presence or absence of NaHCO_3_, the data are also presented as the pHi recovery rate obtained in control conditions subtracted from those obtained in the presence of Cariporide (Figure 4 and Table 1). Indeed, in the absence of NaHCO_3_, HPDE cells had the highest NHE1 activity compared to CPCs and CSCs, and this was dramatically reduced when cells were perfused with NaHCO_3_-containing Ringers (Figure 4). This set of conditions mimics the cycle of digestive hormone-stimulated secretion of NaHCO_3_ and the subsequent proton release, followed by the resetting of basal conditions. Indeed, the cyclical absence of NaHCO_3_, which could be a particularly recurrent situation especially in the juxtatumoral and tumor tissues, would represent a highly unstable situation for HPDE cells, which are forced to strongly activate NHE1 to restore the homeostasis of pHi.

Both CPCs and CSCs had lower NHE1 activity than HPDE cells (Figure 4), but, similarly to HPDE, the contribution of NHE1 to pHi regulation was maximum on 90M-10C and decreased in the Collagen I-enriched ECMs. Moreover, in 90M-10C NHE1 activity was higher in the CSCs in comparison to the CPCs. Interestingly, NHE1 activity in the cancer cells was less affected by the presence of NaHCO_3_ than in the HPDE cells suggesting that, even if the cancer cells also express Na^+^/HCO_3_^−^ transporters, pHi regulation is mainly mediated by NHE1. These functional data are in line with the data-mining analyses of acid-base transporter expression changes in PDAC showing that PDAC has reduced expression of Na^+^/HCO_3_ transporters and an increased expression of NHE1 [10,17].

## 3. Discussion

The physiology of the exocrine pancreas is quite complex and not yet fully understood. Its involvement in digestion is relevant, due to the secretion of an alkaline, NaHCO_3_ -rich fluid [5,57] that (i) neutralizes the chyme arriving from the stomach and (ii) prevents the aggregation of digestive enzymes in pancreatic lumen [5]. NaHCO_3_ secretion is guaranteed by a coordinated activity of the NHE1 and Na^+^/HCO_3_^−^ co-transporters expressed on the basolateral membrane and the Cl^−^/HCO_3_ exchanger (SLC26), the gastric and non-gastric H^+^/K^+^-ATPases [9], and CFTR [10,58] on the apical membrane of duct cells. Furthermore, the exocrine secretion of the pancreas is cyclical and is maximal during the digestive phase when luminal NaHCO_3_^−^ concentration reaches 150 mM [53] During this phase, ductal cells extrude equal amounts of acid across the basolateral membrane, thus acidifying the pancreatic interstitium while activating all the pHi-regulatory proteins to maintain, despite these continuous acid-base fluxes, pHi homeostasis within the physiological range. Importantly, these alternating waves of interstitial acidic pHe might facilitate the onset of certain types of tumors, where the pre-cancer cells are selected under acidic stress and further progress towards malignancy following their prolonged exposition and adaptation to an acidic pHe [29,59,60,61]. Moreover, while an acidic pHe (pHe 6.7) is now recognized as a hallmark of tumor malignancy and metastasis, it also contributes to genetic instability and metabolic rewiring, synergizing with the more alkaline pHi (from pHi 7.4 to pHi 7.7) to promote tumor development [29,37,38,39,59]. In this respect, the most lethal human pancreatic cancer is PDAC, which develops in small ducts disrupting their normal physiology and acid-base regulation [25,62]. Furthermore, pancreatic cancer cells exhibit reduced expression of the NaHCO_3_ transporters [17] causing defects in NaHCO_3_ secretion [53]. This could probably serve to maintain a higher alkaline pHi [17,19], which is associated with a decrease in cell death and an increase in proliferation [61]. Moreover, the inhibition of the Na^+^/HCO_3_^-^ cotransporter SLC4A4 has been recently demonstrated to attenuate the acidic pHe by accumulating extracellular NaHCO_3_ and reduce in vivo tumor growth and metastasis while restoring antitumor immunity and increasing response to immunotherapy [26].

In addition, PDAC is associated with a dense stroma, ever more enriched by collagen I during PDAC progression [37], which, by hindering vascularity in the tumor microenvironment, exacerbates the typical tumor extracellular acidosis and creates acidic niches where the more aggressive Cancer Stem Cells (CSCs) are supposed to arise and survive [29,59,62]. As a consequence of tumor acidosis, cancer cells remodel the ECM by producing and depositing increasing amounts of collagen I, which stabilize cells under acid stress [63]. However, the role of the ECM composition in the regulation of pHi dynamics in both the normal HPDE cells and in the tumor parenchymal cells (CPCs) and their derived CSCs and its interaction with different amounts of extracellular NaHCO_3_ and pHes are so far still unknown. Here, we first demonstrated by spectrofluorimetric analysis that both cancer cell lines, and especially the CSCs, were more alkaline when grown on 90M-10C and the presence of NaHCO_3_, regardless of the external pHe, and the CSCs were more alkaline than the CPCs (Figure 1B,C). This is in accordance with the CSCs ability to transdifferentiate into a vascular-like phenotype [45], since a more alkaline pHi is required for cell differentiation/trans-differentiation [64,65,66]. Further, when grown on Matrigel, CPCs had enhanced invasive ability, which, together with the CSCs’ vasculogenic mimicry, favors the early invasion of CPCs into the aberrant CSC-derived vasculature during the early stage of PDAC progression [45]

Here, we have also identified the fundamental role of ECM composition in determining and maintaining the reversed acid–base balance of the cancer cells with respect to the normal cells [12]. Indeed, when grown on a Collagen I-rich ECM (70M-30C) at pHe 7.4 and in the presence of external NaHCO_3_, we found that the normal HPDE cells maintained their pHi at the physiological 7.2 value and were always more acidic than the CPCs and the CSCs (Figure 1A). However, during both fasting and PDAC progression, the amount of secreted NaHCO_3_ decreases until it is completely absent. Thus, when perfusing the cells with Ringer without NaHCO_3_, we found an increase in the resting pHi in all three cell lines, with the highest pHi observed in the most aggressive CSCs in the presence of a pHe of either 7.4 or 6.7 (Figure 1C). Moreover, the HPDE cells displayed a higher difference in pHi between NaHCO_3_-containing and NaHCO_3_-free Ringers, compared to the cancer cells and especially the CSCs (Figure 1A,C). Importantly, the highest pHi of the three cell lines observed in the absence of NaHCO_3_ at pHe 7.4 (Figure 1) was correlated to a higher pHi recovery rate from an intracellular acidification (Figure 3A–C), which was extremely rapid in the HPDE cells (Figure 3A). Furthermore, cell perfusion with the NaHCO_3_-containing Ringer slowed the pHi recovery from acid load, especially in HPDE cells (Figure 3A), demonstrating the greater involvement of NaHCO_3_ transporters in pHi regulation in normal cells rather than in cancer cells and supporting the observation that bicarbonate transporters are dysregulated in PDAC [8,17,18].

To investigate the relative contribution of NaHCO_3_ transporters or NHE1 in the pHi recovery after acidification, we performed the experiments by perfusing the cells with Ringer’s solutions containing Cariporide, a specific NHE1 inhibitor. We found that in both normal and cancer cells, Cariporide had a greater inhibitory effect on pHi recovery in NaHCO_3_-free Ringer than in the NaHCO_3_-containing Ringer (Figure 2). Moreover, the very high NHE1 activity in HPDE cells in NaHCO_3_-free Ringer was dramatically reduced in the presence of NaHCO_3_ (Figure 3A). This suggests a compensatory activity between the NaHCO_3_ and Na^+^/H^+^ transporters in normal cells in vivo. During the digestive phase, when the duct cells secrete the alkaline fluid, the same transporters that secrete NaHCO_3_ simultaneously regulate the pHi homeostasis. Instead, when NaHCO_3_ secretion is reduced, HPDE cells strongly activate NHE1 to restore the pHi by H^+^ extrusion. During PDAC progression, the ECM is increasingly enriched with Collagen I [37,65] and cancer cells reduce NaHCO_3_ secretion [17]. In this respect, we found that both the CPCs and the CSCs had the highest pHi (Figure 1B,C), due to the higher NHE1 activity in Matrigel-rich ECM mimicking the early development of PDAC. In line with this, the NHE1 activity was less affected in the cancer cells by the presence of external NaHCO_3_, especially in the CSCs (Figure 3B,C and Figure 4), which supports the data-mining analyses of acid–base transporter expression changes in PDAC showing that advanced PDAC has altered expression of NaHCO_3_ transporters and resulted in an increased expression of NHE1 [8,17,18]. Future experiments will be performed to identify and explore the role of NaHCO_3_ transporters involved in these conditions.

We hypothesize a dynamic model of PDAC progression in which during the initial phase of the tumor development CPCs and CSCs are close to the normal duct HPDE cells and are exposed to their secreted NaHCO_3_-rich fluid while they reside in regions with lower amounts of NaHCO_3_ in the tumor core of the more advanced disease. In line with this model, we found that, when the cells grow on Matrigel, the contemporary presence of Na^+^ and NaHCO_3_ in the extracellular milieu permits pHi recovery due to the activation of both NaHCO_3_ transporters and NHE1, while with higher levels of collagen I, the pHi recovery in those conditions was due almost entirely to NHE1 (Figure 3B,C).

Altogether, these data suggest that the composition of the ECM is sufficient to drive the pH regulatory behavior of the cells towards the presence of an NaHCO_3_ gradient similar to that expected in the tumor independently of the intrinsic oncogenic background. That is, on Matrigel (mimicking the tumor periphery), the cells utilized both transport systems to recover pHi, whereas on the ECM mimicking the tumor core, the cells principally utilized the NHE1. This is line with work in PDAC that TME extrinsic factors can override intrinsic signaling to regulate progression and chemotherapy resistance [66,67] and with the recent work in non-small-cell lung carcinoma demonstrating that ECM composition can determine prognosis and risk independently of the clonal heterogeneity of the tumor [68] and even educate immunoregulatory macrophages in ovarian cancer metastasis [69].

## 4. Materials and Methods

### 4.1. Cell Lines

Human Pancreatic Duct Epithelial cells (HPDE-H6c7) are used as a model for the benign/normal pancreatic ductal epithelium. It is an immortalized epithelial cell line derived from normal human pancreatic duct epithelial (HPDE) cells. This cell line demonstrates near-normal genotype and phenotype of HPDE cells and is wild type for p53 and KRAS genes. HPDE were grown in a mixture of 50% RPMI 1640 (Gibco, Life Technologies, Carlsbad, CA, USA), supplemented with 10% FBS, 100 U/mL penicillin G, 0.1 mg/mL streptomycin and 1% non-essential amino acids 100X solution (Gibco, Life Technologies, Carlsbad, CA, USA) and 50% keratinocyte medium SFM (Gibco, Life Technologies) supplemented with 0.025% bovine pituitary extract and 2.5 mg/L epidermal growth factor (Gibco, Life Technologies, Carlsbad, CA, USA). The Panc1 human pancreatic adenocarcinoma parenchymal (CPC) cell line and their derived Cancer Stem Cells (CSCs), generated as previously described [69], were grown and maintained in standard conditions as previously described [44,45]. The CSCs were selected from the PDAC cell line Panc-1 (the Parental line) and identified by their ability to form anchorage-independent colonies and by their overexpression of common CSC markers [44,45,69]. All cell lines were maintained at 37 °C and in 5% CO_2_.

### 4.2. 3D Organotypic Cultures

Three different mixtures composed of Matrigel Basement Membrane Matrix (Corning Inc., NY, USA) and collagen I (bovine-Gibco, Life Technologies, Carlsbad, CA, USA) were prepared as previously described [43,44,45,46]. Matrigel was diluted to a final concentration of 7 mg/mL in serum-free media, whereas collagen I was diluted to the final concentration of 3 mg/mL in distilled sterile water, PBS 10X (Sigma Aldrich, Burlington, MA, USA) and 0.015 N NaOH. Then, Matrigel and collagen I were mixed at different percentages, 90% Matrigel-10% collagen I (90M-10C), 70% Matrigel-30% collagen I (70M-30C) and 10% Matrigel- 90% collagen I (10M-90C) as described [45]. In all cases, 12mm glass coverslips placed in 24-well cell culture plates were coated with a thick layer of the mixes, approximately 90–150 µM thick. The cell culture plates were then incubated at 37 °C with 5% CO_2_ for 1 h to allow the mixture to solidify, after which 2 × 10^5^ cells/well were seeded on the matrix and incubated at 37 °C with 5% CO_2_.

### 4.3. Spectrofluorimetric Measurements of pHi with and without (w/o) NaHCO_3_ with Cary Eclipse

Cells, cultured on 12 mm coverslips as described above, were incubated with 4 µM 2′,7′-Bis-(2-Carboxyethyl)-5-(and-6)-Carboxyfluorescein, Acetoxymethyl Ester (BCECF-AM, ThermoFisher, Waltham, MA, USA) in the dark for 1 h at room temperature and in air without added CO_2_. The coverslip was then placed inside a perfusion cuvette, where all the solutions were maintained at 37 °C and the cells were perfused at the same rate. Using a Cary Eclipse spectrofluorometer, cells were excited alternatively at 440 and 500 nm, while the BCECF fluorescence emission was collected at 535 nm. The resting pHi was measured with Ringer with and without HCO_3_ at both pHe 6.7 and 7.4 (solution compositions reported in Supplemental Table S1).

Intracellular pH (pHi) was estimated from the ratio of BCECF fluorescence calibrated by using the K+ nigericin method according to the Boron protocol [54]. The cells were incubated with 4 µM BCECF-AM and 5 µM nigericin in a KCl-rich Ringer for 1 h at room temperature. After the incubation, the cells were perfused with KCl Ringer at different pH values (6.7, 7.0, 7.4, and 8.0).

### 4.4. Spectrofluorimetric Measurements of NHE1 Activity

The measure of the NHE1 activity was performed using Ringer with and without NaHCO_3_ at pHe 7.4, according to the Boron protocol [53]. Cells were cultured and loaded with BCECF as described above. Next, 40 mM NH_4_Cl was added to the Ringer and dissociated to NH_3_, which readily crosses the cell membrane and, due to binding to H^+^, leads to a rapid intracellular alkalization. After 7 min, the cells were then perfused with Na^+^-free Ringer, which causes acidification via the reverse NH_3_ extrusion process. The pHi recovery was measured replacing the latter Ringer with Na^+^ Ringer, which reactivates the NHE1. In absence of NaHCO_3_, Na^+^ was substituted with an equimolar amount of Tetramethylammonium Chloride (TMA). In contrast, in Ringer with NaHCO_3_, NaCl was replaced with choline-HCO_3_ (Sigma-Aldrich, Waltham, MA, USA). To estimate the contribution of NHE1 to measured pHi recovery, the specific inhibitor of NHE1, Cariporide, was added to Na^+^ Ringer at 5 µM. The pHi recovery rate is expressed in ∆pHi/min and corresponds to the slope of the theoretical line, obtained by the analysis of the least squares, of 15 pH values taken in sequence every 4 s.

### 4.5. Extracellular pH Measurements

Extracellular pH (pHe) measurements were performed 24 h after medium exchange directly in the medium of cultured cells grown to confluence in 96-well plates using single-barreled H^+^-sensitive microelectrodes. ∆pHe was obtained by comparing the pH of medium with and without cells. The measurements were performed in bicarbonate-containing medium. pH microelectrodes were fabricated as described previously for double-barreled microelectrodes [70] but adopting the following modifications. Briefly, single-barreled microelectrodes were constructed from filament-containing aluminum silicate glass tubing of 1.5-mm outer diameter and 1.0 mm inner diameter (Hilgenberg, Malsfeld, Germany). Microelectrodes were pulled in a PE2 vertical puller (Narishige, Tokyo, Japan), silanized in dimethyl-dichloro-silane vapor (Sigma Aldrich) and baked in the oven. Then, the tip of the microelectrode was back-filled with a small amount of the proton ionophore cocktail (Hydrogen Ionophore II, Cocktail A; Sigma) and its shaft was later filled with a buffer solution of pH 7.0. The reference electrode was an Ag/AgCl wire connected to ground. Microelectrodes were calibrated before and after each measurement in NaCl solutions containing a mixture of KH_2_PO_4_ and Na_2_PO_4_ to yield pH values between 6.8 and 7.8 and the measurement was discarded when the measured slope was not within 10% of the calculated slope. The average slope of the electrodes was 58.5 ± 0.3 mV/pH unit (mean ± SEM, n = 19). The pH changes were monitored by lowering perpendicularly the ion-sensitive microelectrode, mounted on a Leitz micromanipulator, in close proximity to the monolayer under oblique (45°) observation through a stereomicroscope (Wild- Heerbrugg, Gais, Switzerland). All measurements were performed with a model FD 223 dual-channel electrometer (World Precision Instruments, New Haven, CT) and recorded on a strip chart recorder (Kipp & Zonen, Delft, Holland).

### 4.6. Statistical Analysis

Statistical analyses were performed using unpaired two-tailed Student’s *t* test and one-way ANOVA with Newman–Keuls as post hoc test with GraphPad Prism 5 (GraphPad Software, version 5), considering significant values to be *p* ≤ 0.05.

## Figures and Tables

**Table 1:** Inhibition of pHi recovery by Cariporide expressed as percentage of control cells in both the presence and the absence of NaHCO_3_.

	HPDE		CPCs			CSCs		
ECMs	90M-10C	70M-30C	90M-10C	70M-30C	10M-90C	90M-10C	70M-30C	10M-90C
**% inhibition NaHCO_3_ (+)**	74 ± 3.10 n = 6	35 ± 12.96 n = 4	89 ± 4.08 n = 3	57 ± 6.87 n = 6	21 ± 5.64 n = 5	71 ± 2.55 n = 3	38 ± 12.29 n = 4	43 ± 5.42 n = 6
**% inhibitionNaHCO_3_ (−)**	81 ± 3.22 n = 10	82 ± 2.16 n = 15	69 ± 4.61 n = 17	76 ± 1.89 n = 8	66 ± 2.61 n = 16	73 ± 3.36 n = 10	56 ± 5.21 n = 17	68 ± 3.69 n = 18

## Data Availability

Not Applicable.

## Supplementary Materials

The following supporting information can be downloaded at: https://www.mdpi.com/article/10.3390/ijms241310632/s1.

## Abbreviations

TME = Tumor Microenvironment; ECM = Extracellular Matrix; CSCs = Cancer Stem Cells; CPCs = Cancer Parenchymal cells; (NHE1) = Na^+^/H^+^ exchanger isoform 1; 90M-10C = 90% Matrigel-10% Collagen I; 70M-30C = 70% Matrigel-30% Collagen I; 10M-90C = 10% Matrigel-90% Collagen I; CAR = cariporide.
