# Supplementary material for: ECM Composition Differentially Regulates Intracellular and Extracellular pH in Normal and Cancer Pancreatic Duct Epithelial Cells
Source: Int J Mol Sci. 2023 Jun 25;24(13):10632. doi: 10.3390/ijms241310632 (PMC10341693; doi:10.3390/ijms241310632)
Supplement: Supplementary file 1 [file ijms-24-10632-s001.zip › ijms-2361143-supplementary.pdf]

***Supplemental Table S1: Composition of Ringers used to measure pHi***

The concentrations are expressed in mM. The pHs of solution w/o bicarbonate are adjusted at 7.4 and 6.7 with NaOH, while the different pHs of Ringer KCl with KOH.

| | **Ringer w $NaHCO_3$ pHe 7.4** | **Ringer w $NaHCO_3$ pHe 6.7** | **Ringer w/o $NaHCO_3$** | **Ringer KCl** |
|---|---|---|---|---|
| **NaCl** | 120 | 138.5 | 135 | 20 |
| **$NaHCO_3$** | 22 | 3.5 | / | / |
| **KCl** | 4.5 | 4.5 | 3 | 110 |
| **$CaCl_2$** | 1 | 1 | 1.8 | 1 |
| **$MgCl_2$** | 1 | 1 | / | / |
| **$MgSO_4$** | / | / | 0.7 | 1 |
| **$KH_2PO_4$** | / | / | 1 | / |
| **Glucose** | 11 | 12 | 11 | 18 |
| **Hepes** | / | / | 20 | 20 |
